# Multiple functions of the vacuole in plant growth and fruit quality

**DOI:** 10.1186/s43897-021-00008-7

**Published:** 2021-06-16

**Authors:** Yu-Tong Jiang, Lu-Han Yang, Ali Ferjani, Wen-Hui Lin

**Affiliations:** 1 Joint International Research Laboratory of Metabolic & Developmental Sciences, School of Life Sciences & Biotechnology, Joint Center for Single Cell Biology, Shanghai Jiao Tong University, Shanghai, 200240 China; 2 Department of Biology, Tokyo Gakugei University, Koganei-shi, 184-8501 Japan

**Keywords:** Vacuole, Biogenesis, Plant growth and development, Protein trafficking, Fruit quality

## Abstract

Vacuoles are organelles in plant cells that play pivotal roles in growth and developmental regulation. The main functions of vacuoles include maintaining cell acidity and turgor pressure, regulating the storage and transport of substances, controlling the transport and localization of key proteins through the endocytic and lysosomal-vacuolar transport pathways, and responding to biotic and abiotic stresses. Further, proteins localized either in the tonoplast (vacuolar membrane) or inside the vacuole lumen are critical for fruit quality. In this review, we summarize and discuss some of the emerging functions and regulatory mechanisms associated with plant vacuoles, including vacuole biogenesis, vacuole functions in plant growth and development, fruit quality, and plant-microbe interaction, as well as some innovative research technology that has driven advances in the field. Together, the functions of plant vacuoles are important for plant growth and fruit quality. The investigation of vacuole functions in plants is of great scientific significance and has potential applications in agriculture.

## Background

The vacuoles of plant cells are multifunctional organelles that display strong plasticity during plant growth and development. Lytic vacuoles (LVs) function as reservoirs for ions and metabolites (e.g., pigments, acids, and toxic substances), and are crucial for general cell homeostasis (Andreev, 2001; Marty, 1999). Vacuoles also play key roles in cellular responses to abiotic and biotic stresses (e.g., microbial invasion) (Miransari, 2014; Nguyen et al., 2015; Swarbreck et al., 2019). In plant vegetative organs, vacuoles act in combination with the cell wall to establish and maintain turgor, the driving force underlying hydraulic stiffness and cell growth (Marty, 1999; Zhang et al., 2014). In seeds and specialized storage tissues, vacuoles serve as storage sites for proteins and soluble carbohydrates. Vacuoles are also reported to modulate stomatal activity (Gao et al., 2010), and to control the localization and transport of key proteins via vacuolar trafficking (Marty, 1999; Offringa & Huang, 2013; Reinhardt et al., 2016; Saini et al., 2017). Thus, vacuoles have several physical and metabolic functions that are essential for plant life.

Vacuole functions are tightly connected with vacuolar proteins, many of which are embedded in the lipid monolayer vacuolar membrane, referred to as the tonoplast. The tonoplast is an important physical barrier that separates the acidic vacuolar lumen compartment from the cytoplasm. Tonoplast-specialized proton pumps, channel proteins, ion transporters, and enzymes located in the tonoplast are essential for the normal function of the vacuole.

The biogenesis and function of plant vacuoles have been topics of interest for decades. Advances in live imaging technology have resulted in constant updates to the field of vacuole-related research. For example, recent studies have shed light on the role of the vacuole in plant embryo development and patterning, through regulating cell division in the embryo (Jiang et al., 2019; Kimata et al., 2019). This review summarizes recent advances in research on vacuole biogenesis, technical methods, and the functions of the vacuole in plant growth and fruit quality.

### Popular technology in current vacuole research

Our understanding of the regulatory mechanisms underlying vacuole-mediated control of plant growth and pattern formation remains fragmentary. As the study of vacuoles usually requires focus on deep subcellular level processes, most observations of vacuoles have been performed using seedling roots, which have the advantages of lacking chloroplasts and thick cell walls. Direct observation of vacuoles in vivo is the best way to disentangle their functions in different tissues, organs, and developmental stages; however, it remains technically challenging to study vacuoles in particular tissues and/or organs, such as ovules and embryos, because they are deeply embedded. One approach to solve this problem is to overexpress *Arabidopsis LEAFY COTYLEDON2* (*LEC2*), a gene encoding a key factor in embryo development and somatic embryogenesis*.* Overexpression of *LEC2* triggers the development of embryos in plant leaves, which allows for a relatively clear view of vacuole morphology (Feeney et al., 2013). Although this system cannot perfectly mimic embryonic vacuolar functions, it has greatly facilitated vacuolar marker signal capture.

Technological improvements have also allowed for more detailed investigations of plant vacuoles. First, technology used for vacuole extraction has matured. Vacuoles from different plants can be extracted and enriched independently, which is convenient for further experiments such as proteomics analysis (Robert et al., 2007). Further, the indirect observation of vacuoles and related proteins in plant cells has improved. Laser confocal scanning microscopy (LCSM), which was originally developed to allow live imaging, is often combined with one or more fluorescent trackable markers, such as VAMP7, VHA-a3, or 2S1; dyes including the pH-sensitive agents, BCECF-AM [3′-O-acetyl-2′,7′-bis (carboxyethyl)-4]; neutral red; time-based dyes (FM-64[N-(N-(3-triethylammoniumpropyl)-4-(6-(4-(diethylamino) phenyl) hexatrienyl) pyridinium dibromide)]; or propidium iodide (Tejos et al., 1789). With continuous improvements in microscope hardware and image processing software, spatial Z-axis and three-dimensional (3D) reconstruction on the T-axis have become rapid and convenient (Cui et al., 2019; Viotti et al., 2013). In addition, LCSM-based live imaging is a powerful tool to monitor the effect of acute pharmacological treatments on signal intensity in living systems. High background noise or poor definition can occur in LCSM when the microscope resolution is less than 1 μm, or when the signal is weak or non-specific. Multiple-layer scans of non-staining fluorescence in living cells can cause fluorescence quenching, resulting in unsatisfactory reconstructed 3D images (Viotti et al., 2013).

Sectioning technique is another important factor for vacuole observation. Although it is possible to obtain sections as thin as 50 nm, or even 1 nm, ultrathin sectioning is time consuming, technically difficult, and challenging to apply to large-scale imaging of living samples, in which co-localized signals cannot be distinguished. Currently, 3D tomography, combined with field emission scanning electron microscopy, is frequently used to build 3D structures, where sections are combined into the highest accuracy steric model of tissue cells. This approach can solve the problem of low-resolution LCSM (Kalinowska et al., 2015; Kolb et al., 2015; Scheuring et al., 2015).

With the rapid development of fluorescence microscopy, technologies involving single-molecule fluorescence imaging in living cells have gradually been applied to research into plant membrane systems and key proteins; relevant approaches include variable-angle total internal reflection fluorescence microscopy (VA-TIRFM) and fluorescence correlation spectroscopy technologies, among others (Lv et al., 2017; Tsuganezawa et al., 2013; Wang et al., 2015a). VA-TIRFM has high resolution, can be used to track the movement rate, lateral displacement, and movement trajectory of membrane proteins, and is most commonly used to study tonoplast proteins (Lv et al., 2017; Wang et al., 2015a). In summary, continuous technological developments provide new perspectives for vacuole study.

### Biogenesis of different vacuole types

Vacuoles can be divided into two types, according to their main function: LVs and protein storage vacuoles (PSVs) (Marty, 1999; Jiang et al., 2000). LVs are specialized compartments found in almost all vegetative tissues. They are involved in substance transportation, storage, and degradation, similar to the roles of lysosomes in animal cells.

PSVs mainly occur from the late embryonic developmental stage to the seed germination stage, and function to store proteins and important minerals during seed filling (Feeney et al., 2018; Zheng & Staehelin, 2011). The above-mentioned authordemonstrated that LVs and PSVs can be mutually transformed during different biological processes (Feeney et al., 2018; Zheng & Staehelin, 2011). The process of vacuole biogenesis has long been an attractive topic for a broad researcher community.

Vacuole initiation has been one of the most controversial issues in plant biology research over the past half century. Evolutionary studies suggest that the important tonoplast proton pump, vacuole H^+^-ATPase, is derived from the P-type ATPase (H^+^-ATPase of the plasma membrane (PM)) of archaea. In addition, evolutionary analyses indicate that the plant tonoplast has a wide range of origins, and that the proteins positioned on it show strong homology with those in plant cell membrane systems (Axelsen & Palmgren, 1998; Vasanthakumar & Rubinstein, 2020). Studies of vacuole initiation are usually observational, and mainly conducted using LCSM technology, sectioning, and other approaches combined with those technologies.

1. **Lytic vacuole biogenesis**

As mentioned above, vacuoles are the largest membrane-bound organelles and have essential roles in plant growth and development, yet several important questions about the biogenesis and dynamics of LVs remain unanswered. The LV is the main type of vacuole and found in most plant organs and plays an important role in maintaining homeostasis within plant cells. There are two hypotheses regarding the biogenesis of LVs: one is that they originate from the endoplasmic reticulum (ER) (Viotti et al., 2013), and the other that they originate from the Golgi apparatus; these two pathways have been well-described by Cui et al. (Cui et al., 2019).

The ER initiation hypothesis is based on observations from the VHA-a3 (a subunit of the tonoplast proton pump V-ATPase) marker line. This hypothesis was tested by specifically blocking various steps of the vacuolar transport pathway and tracking the VHA-a3 signal and vacuole morphology. In that study, the initiation of LVs, including transportation of the tonoplast proton pump and important lipids, appeared to be independent of key proteins in the vacuolar transport system, Rab5 and Rab7. Moreover, the initiation of LVs did not occur in the region containing the Golgi apparatus. It was postulated that the precursors of LVs form in an area enriched with sterols, directly shed from the ER. At the very beginning, vacuole precursors were empty, subsequently gradually expanding to accommodate highly acidic fluid. This process appears to be related to autophagosomes; however, there is no experimental evidence for the involvement of a typical autophagy process in the initiation of LVs (Viotti et al., 2013). Other studies suggested that VHA-a3 transport depends on the trafficking of the small G protein, Rab5, but is independent of regulation by Rab7, with the VHA-a3 protein finally reaching the tonoplast via the trans-Golgi/early endocytosis (TGN/EE) pathway, which is part of the typical vacuolar transport pathway (Feng et al., 2017; Uemura & Ueda, 2014).

Another hypothesis is that LVs in plant cells are independent and separate from each other. Cui et al. (Cui et al., 2019) found no experimental evidence for a clear connection between the vacuole and other membrane systems; using 3D reconstruction techniques, based on continuous ultra-thin slices, they found that LVs could be initiated from multivesicular bodies (MVBs). Further, they observed that internal small vesicles fused together following induction by the SNARE protein, and the body of fused vesicles gradually enlarged, eventually forming an LV (Cui et al., 2019).

(2) **Biogenesis of protein storage vacuoles**

The PSV is a storage organelle specifically formed during plant seed development that plays a key role in storing nutrients from the seed development stage to the germination stage. The initiation of PSVs varies among species. LVs have been reported to transform into PSVs and vice versa; however, the mechanism underlying this process remains unclear.

Pea (*Pisum sativum*) PSVs form de novo, while those of *Arabidopsis thaliana* form via functional reprogramming of LVs (Feeney et al., 2018; Robinson et al., 1995). Observations of *Arabidopsis* embryos from the late torpedo stage showed that the LVs in embryo cells gradually transformed into PSVs. After seed germination, PSVs rapidly transformed back into LVs (Feeney et al., 2018). It is unlikely that such transformations are governed by the same mechanism in all plants. For example, during the germination of tobacco (*Nicotiana tabacum*) seeds, PSVs in root cells were converted into LVs in two different ways: de novo biogenesis and functional reprogramming genesis. Both types of genesis were observed in epidermal, exodermal, endothelial, and vascular cells (Feeney et al., 2018).

### Vacuole-related trafficking influences the transportation and localization of key proteins

Plant cells have complex inner membrane systems, including ER, Golgi, TGN, EE, vacuoles, and so on. The trafficking of intracellular proteins begins with cargo sorting and the formation of transport vesicles. This process is mediated by SAR/ARF GTPases, coat protein complexes (COPI and COPII), and clathrin (Zhang et al., 2014; Uemura & Ueda, 2014; Fan et al., 2015; Takehiko & Takashi, 2017). After vesicles detach from the donor membrane, effectors/tethers interacting with the protein-specific RAB GTPases or GTPases are transported to the target membrane and fuse with the target membrane to unload the protein. Most important physiological activities in plant cells, including the precise localization of key proteins in the cell, depend on membrane system transport pathways (Zhang et al., 2014; Uemura & Ueda, 2014; Fan et al., 2015; Takehiko & Takashi, 2017). There are three main types of membrane system transport pathway: the secretory pathway, the endocytic pathway, and the lysosomal-vacuolar transport pathway (Zhang et al., 2014; Uemura & Ueda, 2014; Fan et al., 2015; Takehiko & Takashi, 2017). Vacuoles have key roles in the endocytic and lysosomal-vacuolar transport pathways, and LV and PSV have unique regulatory pathways for the transport of different proteins in these processes (Bottanelli et al., 2011; Ebine et al., 2014; Kang & Hwang, 2014).

The distribution of most membrane-localized proteins in the PM in plants does not exhibit polarity, while a few proteins with polar localization are of great significance during plant development. The polar localization of these proteins in PM depends on various molecular mechanisms. For example, proteins such as PEN3 and NIP5;1 rely on the extracapsular subunit, EXO84b, to orient in the abaxial-lateral direction in the PM (Mao et al., 2016). Heterogeneous cell growth depends on the geometric edge-directed transport of proteins within the cell, which requires activation of the RAB11/RABA group member, RABA5c/ARA4 (Kirchhelle et al., 2016). Among proteins with polar localization, the auxin polar transporter, PIN1, has been well-studied in recent years. This protein is synthesized in the rough ER, then passes through the TGN/EE and reaches the PM via the endomembrane system. Several studies have shed light on the dynamics of PIN1, and how it is controlled. Initially, PIN1 is evenly distributed in the PM with no polarity. It is then shed from the PM and recovered by the TGN/EE through clathrin-mediated endocytosis. Some PIN1 re-localizes to the PM through a recycling process, is distributed in a polar manner, and functions as a polar transporter of auxin. Remaining PIN1 is transported to the TGN/EE via endocytosis and then moved to the vacuole for degradation. This PIN1 protein vacuolar transport pathway is regulated by auxin concentration and ubiquitination level (Gälweiler et al., 1998; Friml, 2003; Kleine-Vehn & Friml, 2008; Steinmann & Grebe, 1999; Wiśniewska et al., 2006). Intracellular auxin levels that are too high or too low induce PIN proteins to enter the vacuolar degradation pathway following endocytosis. Mono-ubiquitination induces PIN endocytosis; however, poly-ubiquitination of a lysine residue of the hydrophilic ring induces their degradation within the vacuole after endocytosis and transport (Offringa & Huang, 2013; Saini et al., 2017; Dhonukshe et al., 2015; Huang et al., 2010; Kim & Bassham, 2011; Kleine-Vehn et al., 2009; Leitner et al., 2012). When PINs are degraded through the vacuolar degradation pathway, cellular microtubules disaggregate via interactions with the associated proteins CLIP-ASSOCIATED PROTEIN (CLASP) and SORTING NEXIN (SNX). This process triggers movement of PIN from the TGN/EE to the vacuole, and the endosome sorting transport complex (ESCRT) then transfers PINs to endosomes for subsequent degradation in the vacuole via recognition of ubiquitination sites (Offringa & Huang, 2013; Saini et al., 2017; Dhonukshe et al., 2015; Huang et al., 2010; Kim & Bassham, 2011; Kleine-Vehn et al., 2009; Leitner et al., 2012). In this way, the vacuolar degradation pathway plays a key role in maintaining PIN levels and auxin homeostasis through regulating PIN metabolism.

**Fig. 1:**
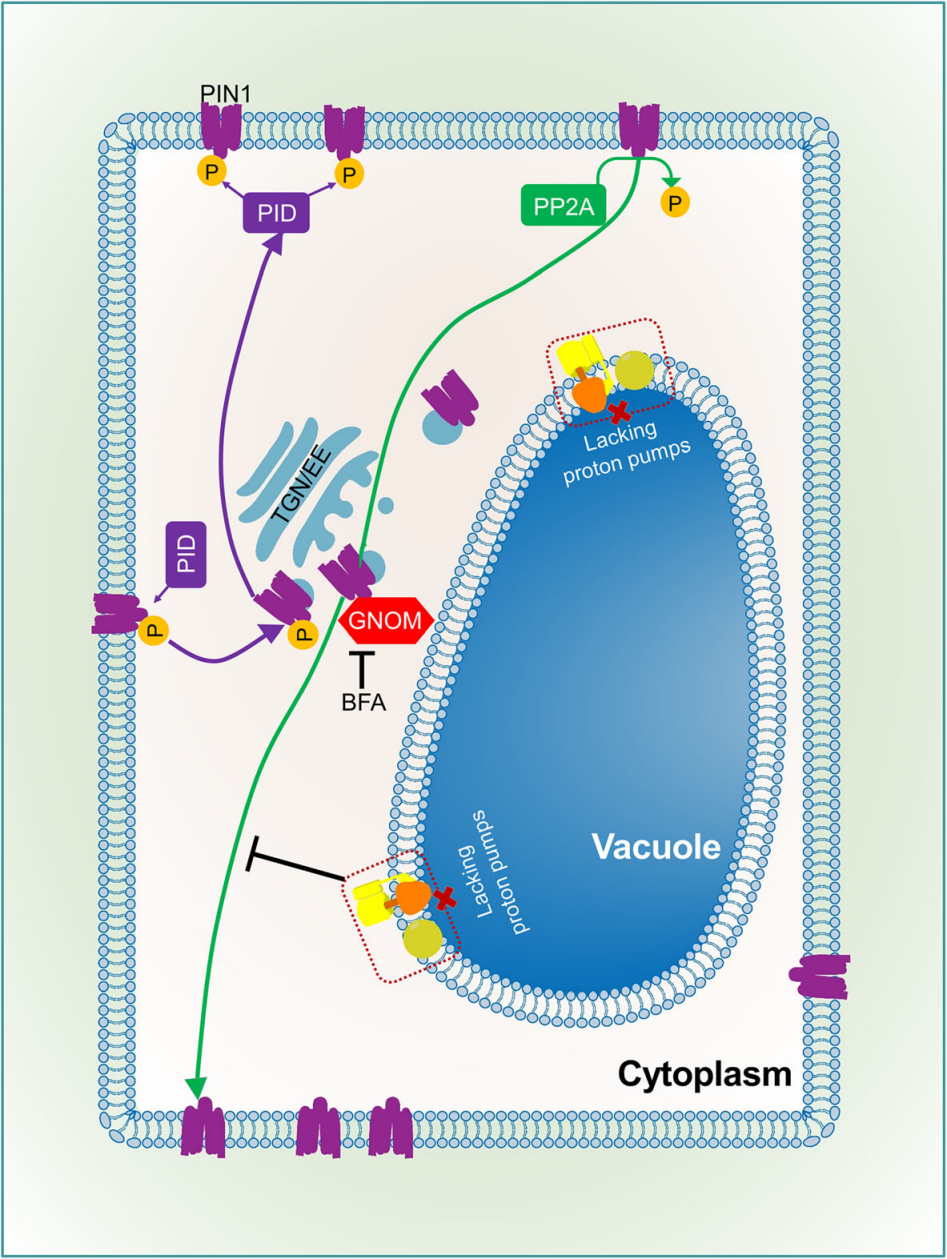
Relationship between tonoplast proton pumps and vesicular trafficking of PIN1 protein. The polar localization of PIN1 protein depends on the vacuolar transport system. The basal-side transport of non-phosphorylated PIN1 protein depends on GNOM, and phosphorylated PIN1 protein moves to the apical region of the membrane. In mutants lacking tonoplast proton pumps, transport of PIN1 to the basal side is inhibited, demonstrating that the tonoplast proton pump affects the vesicular trafficking of PIN1, thereby influencing the polar transport of auxin

In mutants lacking vacuolar proton pumps, the level and distribution of auxin and PIN1 proteins are dramatically affected during embryo and seedling development, and this is tightly connected with abnormal number, size, and shape of vacuoles. PIN1 in the mutant background is insensitive to Brefeldin A treatment, suggesting that PIN1 vesicular trafficking may be defective in the *vap3* background, resulting in abnormal PIN1 polar localization and auxin distribution (Fig. 1) (Jiang et al., 2019).

### Vacuole functions in plant growth and fruit quality

1. **Basic storage function of vacuoles**

As the largest organelle in mature plant cells, the vacuole exhibits complex and diverse functions. First, as a closed compartment, vacuoles can store free amino acids, sugars, and ions. They can also transport key molecules through specific channel proteins on the tonoplast. In addition, tonoplast aquaporins participate in long-distance water transport, and enhance resistance to abiotic stresses, such as drought and flooding (Srivastava et al., 2014). Stomata are the ultimate gas exchange gate in plants, and their morphology changes depending on vacuole water content (Reinhardt et al., 2016; Chrispeels & Daniels, 1997; Footitt et al., 2019).

Secondary metabolism and secondary metabolites are typical characteristics of plants and some microorganisms, and are the result of adaptation to the external environment during evolution. Most secondary metabolites are produced in the cytoplasm. Since some metabolites are toxic to even the plant itself, they are preferentially stored inside vacuoles, where they are isolated from other cellular compartments. Vacuolar-related secondary metabolic processes are widely involved in plant growth and development. The vacuole undergoes regular changes in growth and morphology during the production and secretion of colored nectar, which contains secondary metabolites, such as alkaloids, terpenes, and cyclic olefin ether glycosides (Davies et al., 2005; Fahn, 2010). This process helps to attract pollinators for cross-pollination of plants (Davies et al., 2005; Leshem et al., 2007). During the pollination process, correct guidance of the pollen tube to the micropyle also depends on growth and movement of the vacuole in the correct direction (Ju & Kessler, 2020).

(2) **Vacuole-related cell growth and pattern formation**

The effect of the vacuole on cell growth under the action of auxin is a research hotspot that has been well-summarized by Kaiser and Scheuring (Kaiser & cheuring, 2020). The acid-growth theory proposes that auxin activates the PM H^+^-ATPase, leading to acidification of the apoplast and the cell wall. This activates pH-responsive non-enzymatic proteins, ultimately resulting in xyloglucan sliding, which triggers cell wall loosening (Cosgrove, 2000; McQueen-Mason et al., 1992). Subsequently, cell elongation is achieved by vacuole swelling through water uptake and deposition of new cell wall material (McQueen-Mason et al., 1992; Barbez et al., 2017). Hence, the process of cell elongation relies on the fine tuning of auxin signaling and precise changes in vacuole morphology (Barbez et al., 2017).

**Fig. 2:**
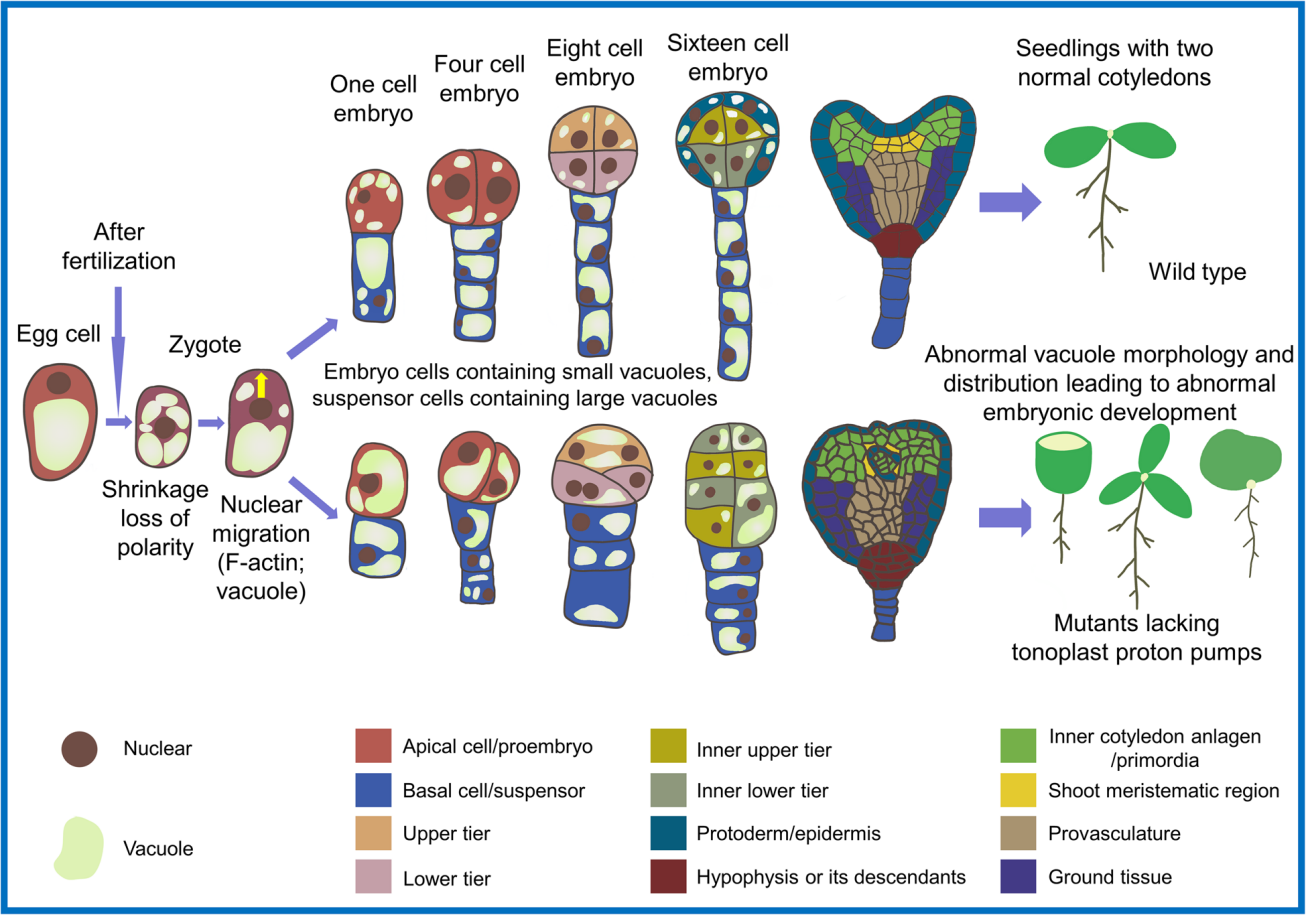
Influence of vacuoles on cell division and morphology of plant embryos and seedlings. After fertilization, the zygote shrinks and loses polarity, and then re-establishes polarity under the action of F-actin and vacuoles. The morphology and distribution of vacuoles in embryo cells and suspensor cells are very important for polarity establishment, embryo patterning, and cotyledon initiation

Vacuole distribution has an essential role in embryonic development and pattern formation. Studies on *Arabidopsis* embryo development revealed the dynamics of the large vacuole in the basal part of the mature egg cell. The volume of the large vacuole immediately decreases after the fertilized egg shrinks (Jensen, 1968; Mayer et al., 1993; Suzuki et al., 1992), leading to loss of polarity of the zygote. Subsequently, the zygote nucleus moves to one end via the action of F-actin. The zygote continuously grows and the polar distribution of the vacuole is re-established in the basal part of the zygote. After the first unequal division of the zygote, a small apical cell and a large basal cell form. At this point, there are several small vacuoles in the dense cytoplasm of the apical cell and a large vacuole in the basal cell (Kimata et al., 2019; Kimata et al., 2016). Very recent work has shown that the morphology and distribution of vacuoles are critical for cell division and pattern formation of the embryo in the early stage of development. Mutants lacking vacuolar proton pumps (namely V-ATPase and V-PPase) showed severe disruption of vacuole morphology and distribution in early embryos (Jiang et al., 2019). Compared with wild-type embryos, mutants formed bigger vacuoles in apical cells and smaller vacuoles in basal cells, leading to an aberrant pattern of embryonic cell division (Fig. 2).

(3) **Contribution of vacuoles to fruit quality**

**Fig. 3:**
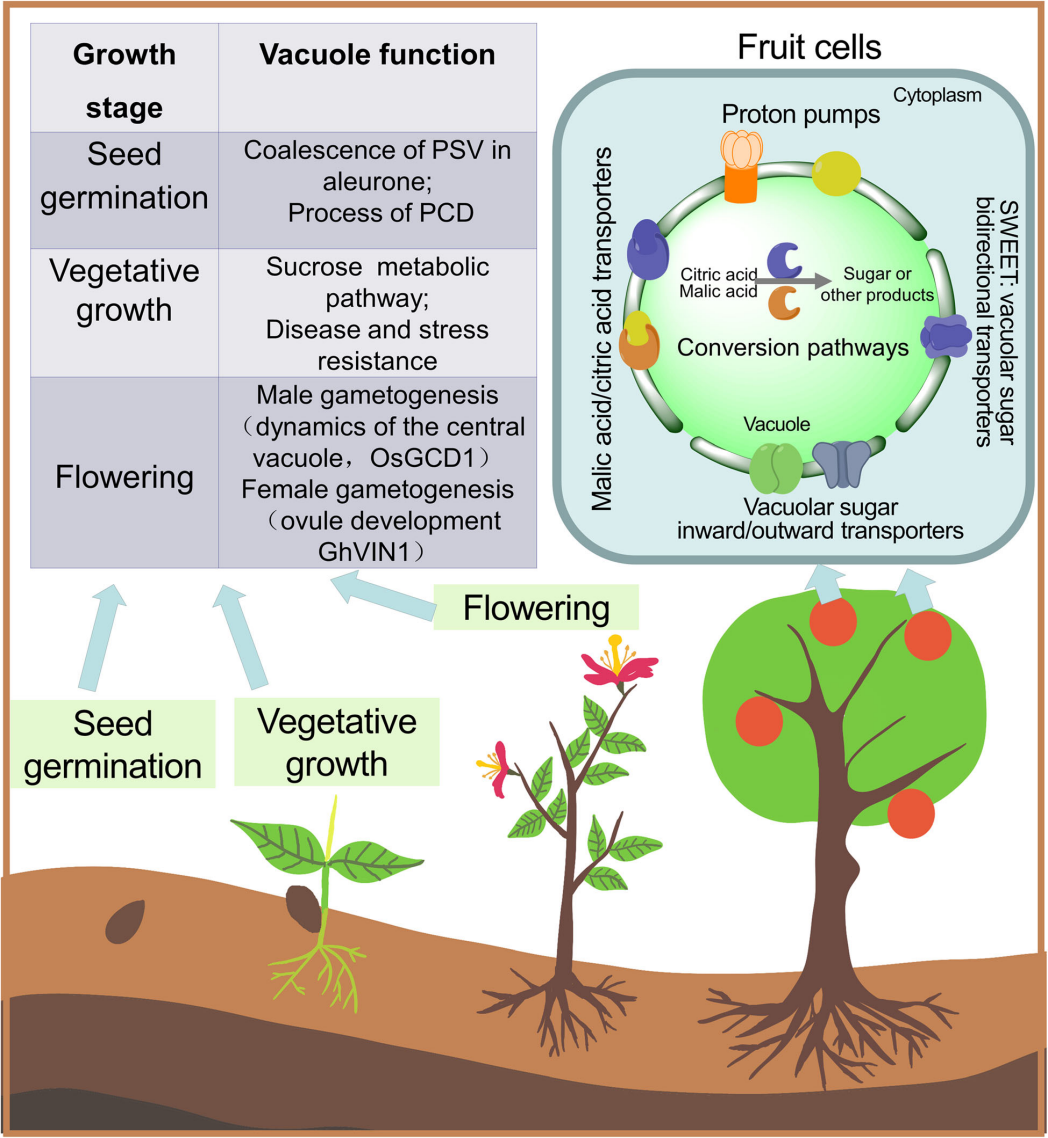
Diverse roles of vacuoles in influencing crop quality. The vacuole is important in many aspects of crop growth, including seed germination, vegetative growth, flowering, and fruit development

Vacuoles are closely related to plant gametophyte development and fertilization. The position of the vacuole plays a key role in the development of crop sperm cells. In rice, OsGCD1 (GAMETE CELLS DEFECTIVE1) dysfunction changes the dynamics of the central vacuole. This leads to incorrect positioning of the male gametophyte, which ultimately affects pollen development and disrupts pollen germination (Huang et al., 2018). The vacuolar invertase, GhVIN1, in cotton (*Gossypium hirsutum*) plays a key role in the timing of pollen release and the normal accumulation of nutrients, such as starch, in the female gametophyte (Wang & Ruan, 2016). GhVIN1 mediates hexose signal transduction and regulates the early differentiation of cotton fibers from the ovule epidermis and their subsequent elongation (Fig. 3) (Wang & Ruan, 2016).

Vacuoles play a key role in seed development. The endosperm and aleurone layer are tissues unique to the seeds of cereal crops (Fath et al., 2000). The endosperm is mainly responsible for storing nutrients, such as proteins and lipids. The aleurone layer wraps around the endosperm tissue of cereal seeds, but is morphologically and biochemically distinct from it. As the only viable tissue after seed maturation, the aleurone layer is responsible for secreting key enzymes. An increase in vacuolization is followed by programmed cell death (PCD), which releases nutrients and enzymes to promote seed germination (Fath et al., 2000; Fath et al., 2010; Pennell & Lamb, 1997). The vacuole is an extremely important organelle in this process. During seed germination, polymers are rapidly hydrolyzed in the PSV lumen by pre-existing enzymes. Gradual fusion of LVs releases key minerals and amino acids to fuel seed germination. This transformation process is promoted by gibberellin and inhibited by abscisic acid (ABA) (Fath et al., 2000; Pennell & Lamb, 1997). The nutrients released from vacuoles are used by the embryo and trigger the PCD process in rice aleurone layer cells. Tonoplast intrinsic proteins in barley (*Hordeum vulgare*) help to prevent the aggregation of small PSVs in aleurone cells (Lee et al., 2015). In barley, ABA was found to induce *HvTIP3; 1* transcription and prevent PSV fusion (Lee et al., 2015). There are two main types of vacuole fusion during the PCD of aleurone cells in rice. The first type is when membranes of multiple small vacuoles fuse to generate large vacuoles. The second type is when large vacuoles engulf small ones, which then rupture inside the large vacuoles and release their contents (Zheng et al., 2017). In this process, vacuolar processing enzyme (VPE) promotes tonoplast fusion and accelerates PCD. Rice OsVPE3 is also involved in the regulation of leaf width and guard cell length (Fig. 3) (Lu et al., 2016).

The taste and quality of fruits are important issues in horticulture research. Vacuoles are the main storage compartments for flavor-related substances, such as sugars and acids (Shiratake & Martinoia, 2007). Vacuolar invertase (VIN or VI) in the vacuole can hydrolyze sucrose into glucose and fructose (Wang et al., 2015b). Both tonoplastic transporters and some hexose metabolic enzymes in the vacuole lumen, can catalyze the conversion of certain substances. Sugar transferases at the tonoplast can be classified as monosaccharide transporters, sucrose transporters SUC/SUT (Sucrose Carrier /Sucrose Transporter), or SWEET (Sugars Will Eventually be Exported Transporters) transporters (Fig. 3) (Feng et al., 2015; Martinoia et al., 2012). The malate transporter and malate ion channels at the tonoplast help to move malic acid and citric acid across the tonoplast. During this process, the tonoplast proton pumps transport hydrogen ions to generate the primary electromotive force; this activity is closely related to fruit flavor. Inhibition of the V-ATPase A subunit in ‘Micro-Tom’ tomato fruit results in significant accumulation of sucrose in the fruit (Amemiya et al., 2005), while the overexpression or heterologous expression of *MdVHP1* (encoding V-PPase) can significantly promote the accumulation of malic acid in apple callus and tomato fruit (Yao et al., 2011). Grapevine (*Vitis vinifera* L.) is a major cultivated fruit crop worldwide. The processes involved in the induction of grape berry ripening have been intensively investigated, with particular focus on the vacuole, since it occupies more than 99% of the total intracellular volume in grape berry (Storey, 1987). The hydrolytic activities of V-PPase and V-ATPase increase throughout development, but especially during ripening, and this process is controlled at both the transcriptional and protein levels (Terrier et al., 2001). The vacuolar acid invertase, PbrAc-Inv1, which is located in the tonoplast of “Fengshui” pears, participates in sucrose hydrolysis and affects the sugar composition and taste of pear fruits. PbrII5 is located in the vacuole lumen and inhibits the activity of PbrAc-Inv1 by combining with it to form an inactive complex and inhibiting the activity of vacuolar acid invertase, thereby reducing sucrose hydrolysis (Ma et al., 2020).

### Involvement of vacuoles in plant stress responses

Horticultural crops are a major source of food, feed, and fuel, and their yields and qualities are related to their ability to cope with fluctuations in the environment. Stress is a major factor that affects crop productivity. Higher plants are often exposed to biotic stress (pathogen invasion) and/or abiotic stress (e.g., salt stress, temperature stress). In this regard, vacuoles are key organelles in maintaining ion homeostasis and stabilizing the intracellular environment. Thus, vacuoles help plants to cope with environmental fluctuations, particularly water scarcity (Lobell et al., 2014).

1. **Vacuole functions in response to abiotic stress**

Transporters at the tonoplast and proteins in the vacuole lumen are vital for tolerance to abiotic stress. The two types of tonoplastic proton pumps, V-ATPase and V-PPase, pump protons from the cytoplasm into the vacuole and maintain relative pH stability in the vacuole, cytoplasm, and other organelles (Ferjani, 2011; Kriegel et al., 2015). V-ATPase and V-PPase have closely related functions, with respect to stress tolerance. Overexpression of V-PPase leads to an enhanced electrochemical gradient across the tonoplast, increased transport and accumulation of toxic ions in the vacuole, and enhanced salt stress tolerance in transgenic tobacco (Li et al., 2017) and creeping bentgrass (*Agrostis stolonifera* L.) (Li et al., 2010); enhanced drought and salt stress tolerance in *Arabidopsis* (Gamboa et al., 2013); and greater drought tolerance in maize (Wang et al., 2016). V-ATPase is important for the development of mung bean (*Vigna radiata*) under cold stress (Kuo et al., 1999; Shahram et al., 2018).

Other membrane transporters, such as the tonoplast Na^+^/H^+^ antiporter, NHX (Na^+^/H^+^ exchanger), use the transmembrane electrochemical potential gradient generated by V-ATPase and V-PPase to sequester toxic Na^+^ in the vacuole, thereby reducing its harmful effects on cells. In addition to tonoplastic transporters, proteins in the vacuole lumen are critical for plant stress resistance (Heven & Salil, 2018; Yokoi et al., 2002). For example, MPK6, a member of the mitogen-activated protein kinase family, up-regulates transcription of *VPE* (encoding vacuolar processing enzyme), and plays an important role in heat shock-induced PCD (Li et al., 2012; Ye et al., 2013).

(2) **Vacuole functions in response to biotic stress**

**Fig. 4:**
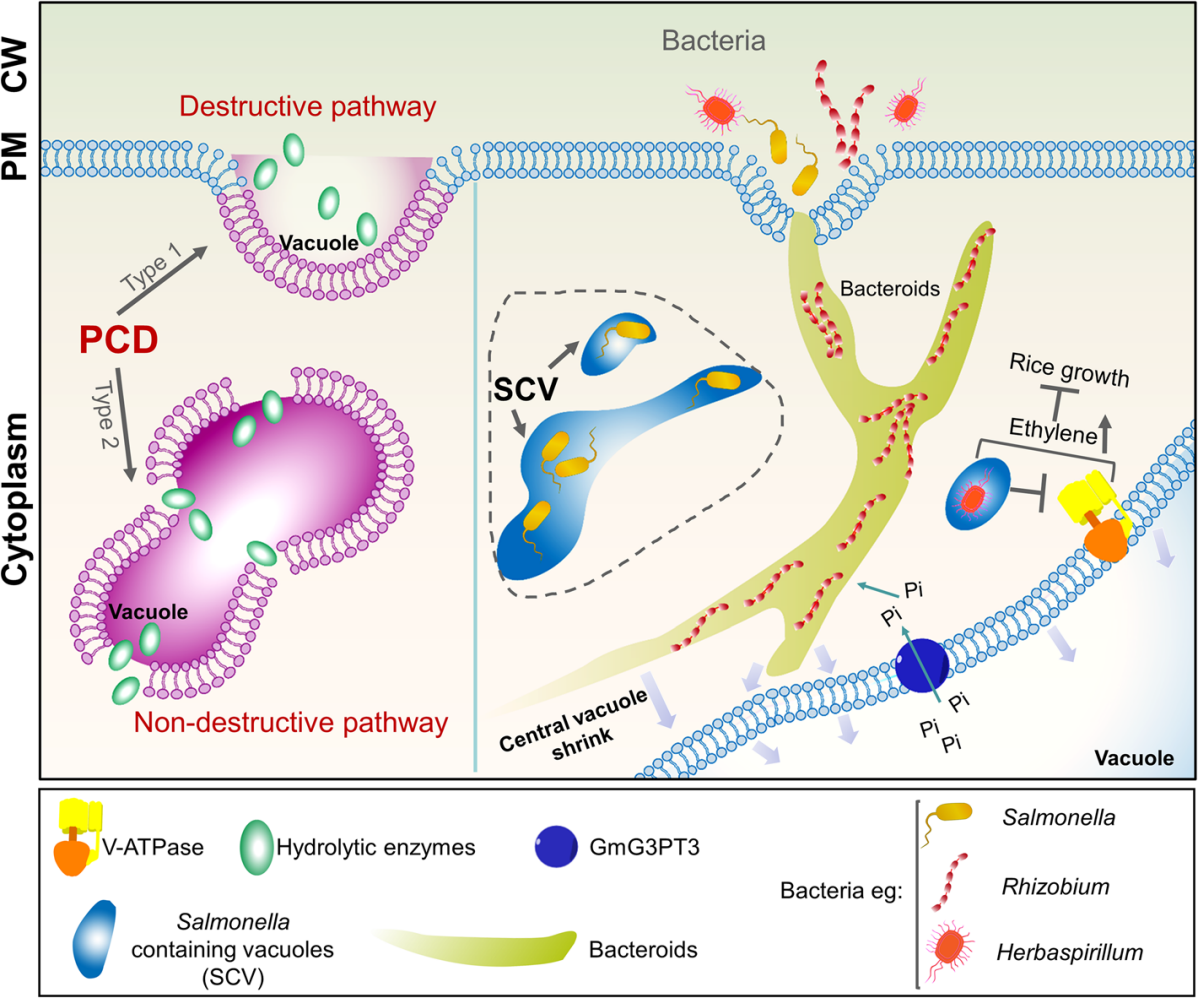
Roles of the vacuole in plant responses to biotic stresses. Plant vacuoles have important roles in resistance to microbial invasion. They mediate two types of programmed cell death (PCD) to eliminate microorganisms from plant cells. Some microorganisms can avoid these elimination strategies and change the morphology and function of plant cells. This model includes information about the PCD process and three types of bacteria

Vacuoles are also important in resistance against microbial infection. Plant-microbe interactions are complex, and include parasitic, antagonistic, and mutually beneficial symbiotic relationships, which have been described in detail previously (Dickman & Fluhr, 2013). During these processes, the vacuole is an important mediator of microbial infection. Bacteria and pathogens must stay inside the vacuole of eukaryotes to be isolated from the cytosolic phagocytic system and lysosomes. For example, the pathogen, *Herbaspirillum rubrisubalbicans*, negatively affects rice plant growth by suppressing V-ATPase activity and increasing ethylene content (Fig. 4) (Valdameri et al., 2017). *Salmonella* establishes a *Salmonella*-containing vacuole (SCV) through membrane remodeling, actin rearrangement, microtubule movement, and adjustment of the autophagy system (Fig. 4) (Steele-Mortimer, 2008). In this way, it is protected from host defenses and can control various processes after entering the cell. *Salmonella* can invade *Arabidopsis* using the same infection strategies with which it infects humans and its broad host range (Huang et al., 2016; Schikora et al., 2011). This finding greatly impacted the study of vacuole function. Although this research is limited to date, we have included a summary in the model diagram, denoted by a dotted line (Fig. 4). *Rhizobium* coexist with legume cells in the form of bacteroids and help plants to fix nitrogen. After bacteroid invasion, the original central vacuole in the plant cell shrinks to make space for the resident bacteroid. Transporters in the tonoplast also provide nutrients for the bacteroid (Gibson et al., 2008; Jones et al., 2007). For example, the sugar-phosphate/anion anti-porter, GmG3PT3, which is located in the tonoplast, participates in inorganic phosphate transport from vacuole to cytoplasm and affects the distribution of phosphorus in nodules (Chen et al., 2019; Li et al., 2018).

Plant cells restrict the spread of pathogens via the hypersensitive response, which involves PCD (Wu & Jackson, 2018). During this process, enzymes with caspase activity alter vacuole morphology and tonoplast structure (Hara-Nishimura & Hatsugai, 2011). Destructive PCD occurs due to tonoplast collapse, which releases vacuolar hydrolases into the cytoplasm, resulting in rapid and direct cell death. This process of cell destruction can effectively eliminate viruses that proliferate in the cytoplasm. In non-destructive PCD, the fusion of the vacuole and PM is triggered in a proteasome-dependent manner. This results in the discharge of vacuolar defense proteins into the extracellular space where bacteria are located (Fig. 4) (Hara-Nishimura & Hatsugai, 2011).

### Concluding remarks

The vacuole is a specific and extremely important organelle in plant cells. Vacuole initiation is related to the evolutionary history of species. Some fungi, bacteria, and protists have vacuoles or analogous organelles. Changes in the morphology, distribution, and function of the vacuole during cell proliferation and budding can provide crucial clues about evolution. The vacuole stores nutrients in cells, and its contents determine the color of cells and tissues and the turgor pressure of the cell. Tonoplast proteins are involved in intracellular ion transport, pH regulation, and vacuole transport pathways.

Studies on vacuole initiation have focused on the dynamics of the vacuole cavity and the transport and re-localization of vacuole-related proteins. How vacuoles originate remains a matter of debate, hence there is an urgent need to obtain experimental evidence supporting or opposing the various hypotheses that have been proposed. The hypothesis of direct initiation from the ER proposes that proteins on the tonoplast reach the vacuole from the ER, without passing through protein transport pathways (the Golgi apparatus) (Uemura & Ueda, 2014; Lupanga et al., 2020). Are there other quality control systems for such proteins? Are they produced in a functionally mature form? When do the contents of the vacuole become acidic? These questions warrant further exploration. The hypothesis that the vacuole is derived from the Golgi proposes that an acidic state exists from the beginning, and that the transport pathway of tonoplast proteins differs from that proposed in the ER hypothesis. Vacuoles may form in multiple ways. For example, observations have suggested that vacuoles originate from MVBs and separate from one another; however, studies on tubular vacuoles suggest that not all vacuoles develop from MVBs. Vacuole initiation may differ depending on plant cell functions. Therefore, there may be other, as-yet unidentified, pathways involved in vacuole initiation. The storage function of plant vacuoles is the basis of plant secondary metabolism, while the distribution of vacuoles also affects plant growth, development, and pattern formation.

Plant products have great impact on human life, and the fruit quality of edible plants is closely related to nutrition intake. Most proteins important for transport and conversion of sugar and acid in fruits are located in the tonoplast and vacuole lumen. The function and activity of these proteins are major determinants of fruit taste and nutrition; however, there has been limited research on the function and regulatory mechanisms of those proteins in different fruits to date. With technological improvements in vacuole extraction methods and the establishment of a vacuole multi-omics database, the key proteins and core regulatory factors underlying the transportation and conversion of sugars and acids in fruit vacuoles will be further explored, and are expected to reveal the regulatory mechanisms underlying the accumulation of sugar and acid in fruit.

Plants tolerate various stresses (including abiotic and biotic stress) in different environments by changing their metabolic processes, which can reduce quality and decrease yield. Vacuoles are also crucial in plant resistance to abiotic stress or bacterial invasion. Although a variety of bacteria and fungi can infect plants, only a few (partial *rhizobia*, nitrogen-fixing bacteria, etc.) can actually enter plant cells and form symbiotic relationships with plants (otherwise, they cause disease). During this process, the central vacuole provides growth space for the microbe, while transporters in the tonoplast provide the necessary ions; however, vacuole functions in the interactions between plants and microorganisms remain unclear. How plant endophytes survive in different organs and plants requires further study. Hence, vacuoles play important roles in multiple physiological processes and the investigation of vacuole functions in plants is of great scientific significance and has potential applications in agriculture.

## Abbreviations

3D: Three-dimensional
ABA: Abscisic acid
ER: Endoplasmic reticulum
LCSM: Laser confocal scanning microscopy
LVs: Lytic vacuoles
MVBs: multivesicular bodies
PCD: Programmed cell death
PM: Plasma membrane
PSVs: Protein storage vacuoles
TGN/EE: Trans-golgi/early endocytosis
VA-TIRFM: Variable-angle total internal reflection fluorescence microscopy
VPE: Vacuolar processing enzyme

## References

[CR1] Amemiya T, Kanayama Y, Yamaki S, Yamada K, Shiratake K (2005). Fruit-specific V-ATPase suppression in antisense-transgenic tomato reduces fruit growth and seed formation. Planta..

[CR2] Andreev IM (2001). Functions of the vacuole in higher plant cells. Russ J Plant Physl+.

[CR3] Axelsen KB, Palmgren MG (1998). Evolution of substrate specificities in the P-type ATPase superfamily. J Mol Evol.

[CR4] Barbez E, Dünser K, Gaidora A, Lendl T, Busch W (2017). Auxin steers root cell expansion via apoplastic pH regulation in Arabidopsis thaliana. Proc Natl Acad U S A.

[CR5] Bottanelli F, Foresti O, Hanton S, Denecke J (2011). Vacuolar transport in tobacco leaf epidermis cells involves a single route for soluble cargo and multiple routes for membrane cargo. Plant Cell.

[CR6] Chen L, Qin L, Zhou L, Li X, Chen Z, Sun L, Wang W, Lin Z, Zhao J, Yamaji N, Ma JF, Gu M, Xu G, Liao H (2019). A nodule-localized phosphate transporter GmPT7 plays an important role in enhancing symbiotic N (2) fixation and yield in soybean. New Phytol.

[CR7] Chrispeels MJ, Daniels MJ (1997). Aquaporins and water transport across the tonoplast. Adv Bot Res.

[CR8] Cosgrove DJ (2000). Expansive growth of plant cell walls. Plant Physiol Biochem.

[CR9] Cui Y, Cao W, He Y, Zhao Q, Wakazaki M, Zhuang X, Gao J, Zeng Y, Gao C, Ding Y, Wong HY, Wong WS, Lam HK, Wang P, Ueda T, Rojas-Pierce M, Toyooka K, Kang BH, Jiang L (2019). A whole-cell electron tomography model of vacuole biogenesis in Arabidopsis root cells. Nature Plants.

[CR10] Davies KL, Stpiczyńska M, Gregg A (2005). Nectar-secreting floral stomata in Maxillaria anceps Ames & C. Schweinf. (Orchidaceae). Ann Bot.

[CR11] Dhonukshe P, Huang F, Galvan-Ampudia CS, Mahonen AP, Kleine-Vehn J, Xu J (2015). Plasma membrane-bound AGC3 kinases phosphorylate PIN auxin carriers at TPRXS(N/S) motifs to direct apical PIN recycling. Development..

[CR12] Dickman MB, Fluhr R (2013). Centrality of host cell death in plant-microbe interactions. Annu Rev Phytopathol.

[CR13] Ebine K, Inoue T, Ito J, Ito E, Uemura T, Goh T, Abe H, Sato K, Nakano A, Ueda T (2014). Plant vacuolar trafficking occurs through distinctly regulated pathways. Current Biology Cb.

[CR14] Fahn A (2010). Secretory tissues in vascular plants. New Phytol.

[CR15] Fan L, Li R, Pan J, Ding Z, Lin J (2015). Endocytosis and its regulation in plants. Trends Plant Sci.

[CR16] Fath A, Bethke P, Lonsdale J, Meza-Romero R, Jones R (2000). Programmed cell death in cereal aleurone. Plant Mol Biol.

[CR17] Fath A, Bethke PC, Jones RL (2010). Barley aleurone cell death is not apoptotic: characterization of nuclease activities and DNA degradation. Plant J.

[CR18] Feeney M, Frigerio L, Cui Y, Menassa R (2013). Following vegetative to embryonic cellular changes in leaves of Arabidopsis overexpressing LEAFY COTYLEDON2. Plant Physiol.

[CR19] Feeney M, Kittelmann M, Menassa R, Hawes C, Frigerio L (2018). Protein storage vacuoles originate from remodeled preexisting vacuoles in Arabidopsis thaliana. Plant Physiol.

[CR20] Feng CY, Han JX, Han XX, Jiang J (2015). Genome-wide identification, phylogeny, and expression analysis of the SWEET gene family in tomato. Gene..

[CR21] Feng QN, Zhang Y, Li S (2017). Tonoplast targeting of VHA-a3 relies on a Rab5-mediated but Rab7-independent vacuolar trafficking route. J Integr Plant Biol.

[CR22] Ferjani A (2011). V-ATPase dysfunction under excess zinc inhibits Arabidopsis cell expansion. Plant Signal Behav.

[CR23] Footitt S, Clewes R, Feeney M, Finch-Savage WE, Frigerio L. Aquaporins influence seed dormancy and germination in response to stress. Plant Cell Environ. 2019;42:2325–39. 10.1111/pce.13561 PMC6767449 30986891

[CR24] Friml J (2003). Auxin transport—shaping the plant. Plant Biol.

[CR25] Gälweiler LGC, Müller A (1998). Regulation of polar auxin transport by AtPIN1 in Arabidopsis vascular tissue. Science..

[CR26] Gamboa MC, Baltierra F, Leon G, Krauskopf E (2013). Drought and salt tolerance enhancement of transgenic Arabidopsis by overexpression of the vacuolar pyrophosphatase 1 (EVP1) gene from Eucalyptus globulus. Plant Physiol Biochem.

[CR27] Gao X, Wang X, Fei R, Jia C, Wang X (2010). Dynamics of vacuoles and actin filaments in guard cells and their roles in stomatal movement. Plant Cell Environ.

[CR28] Gibson KE, Kobayashi H, Walker GC (2008). Molecular determinants of a symbiotic chronic infection. Annu Rev Genet.

[CR29] Hara-Nishimura I, Hatsugai N (2011). The role of vacuole in plant cell death. Cell Death Differ.

[CR30] Heven S, Salil C. Plant endomembrane dynamics: studies of K+/H+ antiporters provide insights on the effects of pH and ion homeostasis. Plant Physiol. 2018;177(3):875–95. 10.1104/pp.18.00142 PMC6053008 29691301

[CR31] Huang F, Zago MK, Abas L, van Marion A, Galvan-Ampudia CS, Offringa R (2010). Phosphorylation of conserved PIN motifs directs Arabidopsis PIN1 polarity and auxin transport. Plant Cell.

[CR32] Huang M, Wu Y, He P (2016). Advances in humans and animals opportunistic pathogens from environment infecting plants by crossing kingdoms. Acta Microbiol Sin.

[CR33] Huang X, Peng X, Xie F, Mao W, Chen H, Sun MX. The stereotyped positioning of the generative cell associated with vacuole dynamics is not required for male gametogenesis in rice pollen. New Phytologist. 2018;218(2):463–9. 10.1111/nph.15038 29424430

[CR34] Jensen WA (1968). Cotton embryogenesis: the zygote. Planta Planta.

[CR35] Jiang L, Phillips TE, Rogers SW, Rogers JC (2000). Biogenesis of the protein storage vacuole crystalloid. J Cell Biol.

[CR36] Jiang YT, Tang RJ, Zhang YJ, Xue HW, Ferjani A, Luan S, et al. Two tonoplast proton pumps function in Arabidopsis embryo development. New Phytologist. 2020;225:1606–17 10.1111/nph.16231 31569267

[CR37] Jones KM, Kobayashi H, Davies BW, Taga ME, Walker GC (2007). How rhizobial symbionts invade plants: the Sinorhizobium-Medicago model. Nat Rev Microbiol.

[CR38] Ju Y, Kessler SA (2020). Keeping pollen tubes on track. Nature Plants..

[CR39] Kaiser S, Scheuring D (2020). To Lead or to follow: contribution of the plant vacuole to cell growth. Front Plant Sci.

[CR40] Kalinowska K, Nagel MK, Goodman K, Cuyas L, Isono E. Arabidopsis ALIX is required for the endosomal localization of the deubiquitinating enzyme AMSH3. Proceedings of the National Academy of Sciences of the United States of America. 2015;112(40). 10.1073/pnas.1510516112 PMC4603487 26324913

[CR41] Kang H, Hwang I (2014). Vacuolar sorting receptor-mediated trafficking of soluble vacuolar proteins in plant cells. Plants..

[CR42] Kim SJ, Bassham DC (2011). TNO1 is involved in salt tolerance and vacuolar trafficking in Arabidopsis. Plant Physiol.

[CR43] Kimata Y, Higaki T, Kawashima T, Kurihara D, Sato Y, Yamada T, Hasezawa S, Berger F, Higashiyama T, Ueda M (2016). From the cover: cytoskeleton dynamics control the first asymmetric cell division in Arabidopsis zygote. Proc Natl Acad Sci U S A.

[CR44] Kimata Y, Kato T, Higaki T, Kurihara D, Yamada T, Segami S, Morita MT, Maeshima M, Hasezawa S, Higashiyama T, Tasaka M, Ueda M (2019). Polar vacuolar distribution is essential for accurate asymmetric division of Arabidopsis zygotes. Proc Natl Acad Sci U S A.

[CR45] Kirchhelle C, Chow CM, Foucart C, Neto H, Stierhof YD, Kalde M, Walton C, Fricker M, Smith RS, Jérusalem A, Irani N, Moore I (2016). The specification of geometric edges by a plant Rab GTPase is an essential cell-patterning principle during organogenesis in Arabidopsis. Dev Cell.

[CR46] Kleine-Vehn J, Friml J (2008). Polar targeting and endocytic recycling in auxin-dependent plant development. Annu Rev Cell Dev Biol.

[CR47] Kleine-Vehn J, Huang F, Naramoto S, Zhang J, Michniewicz M, Offringa R, Friml J (2009). PIN auxin efflux carrier polarity is regulated by PINOID kinase-mediated recruitment into GNOM-independent trafficking in Arabidopsis. Plant Cell.

[CR48] Kolb C, Nagel MK, Kalinowska K, Hagmann J, Ichikawa M, Anzenberger F, Alkofer A, Sato MH, Braun P, Isono E (2015). FYVE1 is essential for vacuole biogenesis and intracellular trafficking in Arabidopsis. Plant Physiol.

[CR49] Kriegel A, Andres Z, Medzihradszky A, Kruger F, Scholl S, Delang S (2015). Job sharing in the endomembrane system: vacuolar acidification requires the combined activity of V-ATPase and V-PPase. Plant Cell.

[CR50] Kuo SY, Tzeng CM, Lin WJ, Jiang SS, Pan RL (1999). An essential arginine residue in vacuolar H+-ATPase purified from etiolated mung bean seedlings. Bot Bull Acad Sin Taipei.

[CR51] Lee SE, Yim HK, Lim MN, Yoon I, Kim J, Hwang YS (2015). Abscisic acid prevents the coalescence of protein storage vacuoles by upregulating expression of a tonoplast intrinsic protein gene in barley aleurone. J Exp Bot.

[CR52] Leitner J, Petrasek J, Tomanov K, Retzer K, Parezova M, Korbei B, Bachmair A, Zazimalova E, Luschnig C (2012). Lysine63-linked ubiquitylation of PIN2 auxin carrier protein governs hormonally controlled adaptation of Arabidopsis root growth. Proc Natl Acad Sci U S A.

[CR53] Leshem Y, Melamedbook N, Cagnac O, Ronen G, Nishri Y, Solomon M (2007). Suppression of Arabidopsis vesicle-SNARE expression inhibited fusion of H2O2-containing vesicles with tonoplast and increased salt tolerance. Proc Natl Acad Sci U S A.

[CR54] Li S, Guo J, Guo Q, Mao PC, Tian XX, Lin M. Overexpression of the Iris lactea H^+^ −PPase enhanced drought resistance and salt tolerance of transgenic tobacco. J Grassland Forage Ence. 2017;7:17779.

[CR55] Li X, Lai Y, Qi W, Liao H. The soybean G3P/Pi transporter, GmG3PT3,plays critical roles in root hair growth in response to phosphate starvation. Natl Congress Plant Biol. 2018;19(7):2145.

[CR56] Li Z, Baldwin CM, Hu Q, Liu H, Luo H (2010). Heterologous expression of Arabidopsis H+-pyrophosphatase enhances salt tolerance in transgenic creeping bentgrass (Agrostis stolonifera L.). Plant Cell Environ.

[CR57] Li Z, Yue H, Xing D. MAP Kinase 6-mediated activation of vacuolar processing enzyme modulates heat shock-induced programmed cell death in Arabidopsis. New Phytologist. 2012;195(1):85–96. 10.1111/j.1469-8137.2012.04131.x 22497243

[CR58] Lobell DB, Roberts MJ, Schlenker W, Braun N, Little BB, Rejesus RM, Hammer GL (2014). Greater sensitivity to drought accompanies maize yield increase in the U.S. Midwest. Science..

[CR59] Lu W, Deng M, Guo F, Wang M, Zeng Z, Han N (2016). Suppression of OsVPE3 enhances salt tolerance by attenuating vacuole rupture during programmed cell death and affects stomata development in rice. Rice (N Y).

[CR60] Lupanga U, Rohrich R, Askani J, Hilmer S, Kiefer C, Krebs M, et al. The Arabidopsis V-ATPase is localized to the TGN/EE via a seed plant-specific motif. Elife. 2020;9. 10.7554/eLife.60568. 10.7554/eLife.60568 PMC7717909 33236982

[CR61] Lv X, Jing Y, Xiao J, Zhang Y, Zhu Y, Julian R, Lin J (2017). Membrane microdomains and the cytoskeleton constrain AtHIR1 dynamics and facilitate the formation of an AtHIR1-associated immune complex. Plant J.

[CR62] Ma M, Wang LB, Zhang SL, Guo L, Zhang SL (2020). Acid vacuolar invertase 1 (PbrAc-Inv1) and invertase inhibitor 5 (PbrII5) were involved in sucrose hydrolysis during postharvest pear storage. Food Chem.

[CR63] Mao H, Nakamura M, Viotti C, Grebe M (2016). A framework for lateral membrane trafficking and polar tethering of the PEN3 ATP-binding cassette transporter. Plant Physiol.

[CR64] Martinoia E, Meyer S, De Angeli A, Nagy R (2012). Vacuolar transporters in their physiological context. Annu Rev Plant Biol.

[CR65] Marty F (1999). Plant Vacuoles. Plant Cell.

[CR66] Mayer U, Berleth T, Ruiz RAT, Miséra S, Jürgens G. Pattern Formation during Arabidopsis Embryo Development 1993.

[CR67] McQueen-Mason S, Durachko DM, Cosgrove DJ (1992). Two endogenous proteins that induce cell wall extension in plants. Plant Cell.

[CR68] Miransari M, Hakeem KR, Rehman RU, Tahir I (2014). Plant, mycorrhizal Fungi, and bacterial network. Plant signaling: understanding the molecular crosstalk.

[CR69] Nguyen HT, Park H, Koster KL, Cahoon RE, Nguyen HTM, Shanklin J, Clemente TE, Cahoon EB (2015). Redirection of metabolic flux for high levels of omega-7 monounsaturated fatty acid accumulation in camelina seeds. Plant Biotechnol J.

[CR70] Offringa R, Huang F (2013). Phosphorylation-dependent trafficking of plasma membrane proteins in animal and plant cells. J Integr Plant Biol.

[CR71] Pennell RI, Lamb C (1997). Programmed cell death in plants. Plant Cell.

[CR72] Reinhardt H, Hachez C, Bienert MD, Beebo A, Swarup K, Voss U, et al. Tonoplast aquaporins facilitate lateral root emergence. Plant Physiol. 2016;170(3):1640–54. 10.1104/pp.15.01635 PMC4775129 26802038

[CR73] Robert S, Zouhar J, Carter C, Raikhel N (2007). Isolation of intact vacuoles from Arabidopsis rosette leaf-derived protoplasts. Nat Protoc.

[CR74] Robinson DG, Hoh B, Hinz G, Jeong BK (1995). One vacuole or two vacuoles - do protein storage vacuoles Arise De novo during pea Cotyledon development. J Plant Physiol.

[CR75] Saini K, Markakis MN, Zdanio M, Balcerowicz DM, Beeckman T, De Veylder L (2017). Alteration in auxin homeostasis and signaling by overexpression of PINOID kinase causes leaf growth defects in Arabidopsis thaliana. Front Plant Sci.

[CR76] Scheuring D, Löfke C, Krüger F, Kittelmann M, Eisa A, Hughes L (2015). Actin-dependent vacuolar occupancy of the cell determines auxin-induced growth repression. Proc Natl Acad Sci U S A.

[CR77] Schikora A, Virlogeux-Payant I, Bueso E, Garcia AV, Nilau T, Charrier A, Pelletier S, Menanteau P, Baccarini M, Velge P, Hirt H (2011). Conservation of Salmonella infection mechanisms in plants and animals. PLoS One.

[CR78] Shahram T, Salar FA, Judith R (2018). Biochar and lignite affect H + −ATPase and H + −PPase activities in root tonoplast and nutrient contents of mung bean under salt stress. Plant Physiol Biochem.

[CR79] Shiratake K, Martinoia E (2007). Transporters in fruit vacuoles. Plant Biotechnol.

[CR80] Srivastava AK, Penna S, Dong VN, LSP T. Multifaceted roles of aquaporins as molecular conduits in plant responses to abiotic stresses. Crit Rev Biotechnol. 2014;36(3):389–98. 10.3109/07388551.2014.973367 25430890

[CR81] Steele-Mortimer O (2008). The Salmonella-containing vacuole: moving with the times. Curr Opin Microbiol.

[CR82] Steinmann TGN, Grebe M (1999). Coordinated polar localization of auxin efflux carrier PIN1 by GNOM ARF GEF. Science..

[CR83] Storey R (1987). Potassium localization in the grape berry pericarp by energy-dispersive X-ray microanalysis. Am J Enol Viticulture.

[CR84] Suzuki K, Taniguchi T, Maeda E (1992). Ultrastructure and cleavage pattern of rice [Oryza sativa] proembryos. Jpn J Crop Sci.

[CR85] Swarbreck S, Wang M, Wang Y, Kindred D, Sylvester-Bradley R, Shi W (2019). A roadmap for lowering crop nitrogen requirement. Trends Plant Sci.

[CR86] Takehiko, Kanazawa, Takashi, Ueda. Exocytic trafficking pathways in plants: why and how they are redirected. The New phytologist. 2017. 10.1111/nph.14613 28543308

[CR87] Tejos R, Osorio-Navarro C, Norambuena L (1789). The use of drugs in the study of vacuole morphology and trafficking to the vacuole in Arabidopsis thaliana. Methods Mol Biol.

[CR88] Terrier N, Ageorges A, Abbal P, Romieu C (2001). Generation of ESTs from grape berry at various developmental stages. J Plant Physiol.

[CR89] Tsuganezawa K, Shinohara Y, Ogawa N, Tsuboi S, Tanaka A (2013). Two-colored fluorescence correlation spectroscopy screening for LC3-P62 interaction inhibitors. J Biomol Screen.

[CR90] Uemura T, Ueda T (2014). Plant vacuolar trafficking driven by RAB and SNARE proteins. Curr Opin Plant Biol.

[CR91] Valdameri G, Alberton D, Moure VR, Kokot TB, Kukolj C, Brusamarello-Santos LCC, Monteiro RA, Pedrosa FO, de Souza EM (2017). Herbaspirillum rubrisubalbicans, a mild pathogen impairs growth of rice by augmenting ethylene levels. Plant Mol Biol.

[CR92] Vasanthakumar T, Rubinstein JL (2020). Structure and roles of V-type ATPases. Trends Biochem Sci.

[CR93] Viotti C, Kruger F, Krebs M, Neubert C, Fink F, Lupanga U (2013). The endoplasmic reticulum is the main membrane source for biogenesis of the lytic vacuole in Arabidopsis. Plant Cell.

[CR94] Wang L, Ruan YL (2016). Critical roles of vacuolar Invertase in floral organ development and male and female fertilities are revealed through characterization of GhVIN1-RNAi cotton plants. Plant Physiol.

[CR95] Wang X, Li X, Deng X, Luu D-T, Maurel C, Lin J (2015). Single-molecule fluorescence imaging to quantify membrane protein dynamics and oligomerization in living plant cells. Nat Protoc.

[CR96] Wang X, Wang H, Liu S, Ferjani A, Li J, Yan J, Yang X, Qin F (2016). Genetic variation in ZmVPP1 contributes to drought tolerance in maize seedlings. Nat Genet.

[CR97] Wang Y, Chen J, Feng J, Qin Q, Huang J. Overexpression of a loquat (*Eriobotrya japonica* Lindl.) vacuolar invertase affects sucrose levels and growth. Plant Cell Tissue Organ Cult. 2015b.

[CR98] Wiśniewska JXJ, Seifertová D (2006). Polar PIN localization directs auxin flow in plants. Science.

[CR99] Wu Q, Jackson D (2018). Detection of MAPK3/6 phosphorylation during hypersensitive response (HR)-associated programmed cell death in plants. Methods Mol Biol.

[CR100] Yao YX, Dong QL, You CX, Zhai H, Hao YJ (2011). Expression analysis and functional characterization of apple MdVHP1 gene reveals its involvement in Na+, malate and soluble sugar accumulation. Plant Physiol Biochem.

[CR101] Ye Y, Li Z, Xing D (2013). Nitric oxide promotes MPK6-mediated caspase-3-like activation in cadmium-induced Arabidopsis thaliana programmed cell death. Plant Cell Environ.

[CR102] Yokoi S, Quintero FJ, Cubero B, Ruiz MT, Bressan RA, Hasegawa PM, Pardo JM (2002). Differential expression and function of Arabidopsis thaliana NHX Na+/H+ antiporters in the salt stress response. Plant J.

[CR103] Zhang C, Hicks GR, Raikhel NV (2014). Plant vacuole morphology and vacuolar trafficking. Front Plant Sci.

[CR104] Zheng H, Staehelin LA (2011). Protein storage vacuoles are transformed into lytic vacuoles in root meristematic cells of germinating seedlings by multiple, cell type-specific mechanisms. Plant Physiol.

[CR105] Zheng Y, Zhang H, Deng X, Liu J, Chen H (2017). The relationship between vacuolation and initiation of PCD in rice (Oryza sativa) aleurone cells. Sci Rep.

